# Machine Learning-Based Foreign Object Detection in Wireless EV Charging Using Planar Magnetic Induction Tomography

**DOI:** 10.3390/s26113486

**Published:** 2026-06-01

**Authors:** Abdul Khader Abdul Vahid, Dorian Vargas-Reighley, Benjamin Warrington, Gavin Dingley, Manuchehr Soleimani

**Affiliations:** 1 Electric Green Ltd., Kingston-upon-Thames KT1 3GZ, UK; abdulkhader@electricgreenev.com (A.K.A.V.); dorian.vargas.reighley@electricgreenev.com (D.V.-R.); ben.warrington@electricgreenev.com (B.W.); 2 Engineering Tomography Laboratory (ETL), University of Bath, Bath BA2 7AY, UK; gd310@bath.ac.uk

**Keywords:** wireless power transfer, foreign object detection, electric vehicles, machine learning, magnetic inductance tomography

## Abstract

Wireless power transfer (WPT) systems for electric vehicles require reliable foreign object detection (FOD) mechanisms both during and prior to power transfer to ensure operational safety and efficiency. The primary purpose of this study was to develop a foreign object detection system to ensure that no objects are present in the area of magnetic coupling (between primary and secondary coils) prior to initiating power transfer. Conventional FOD techniques based on impedance, visual light, or thermal monitoring provide limited spatial information and are sensitive to coil misalignment. This paper proposes a machine learning-based FOD approach using a planar Magnetic Inductance Tomography (MIT) sensor array that enables spatial electromagnetic sensing for early detection and localisation of conductive foreign objects. A dataset comprising 17,800 measurement frames was collected using a custom STM32-based data acquisition system in the absence of (prior to) power transfer. Likewise, a dataset comprising 300 sets of measurement frames was collected during power transfer, in which each frame contains 120 electromagnetic sensor readings. This capture methodology coincides with the detection requirements of live WPT systems. Four classification models, including Random Forest, Support Vector Machine, XGBoost, and Multi-Layer Perceptron, were evaluated. To enhance robustness against sensor drift and environmental variations, feature-engineering techniques incorporating statistical, temporal, frequency-domain, and derivative-based features were developed. Experimental results demonstrate high detection accuracy under both controlled and real-world conditions. The proposed approach demonstrates the feasibility of integrating machine learning-based MIT sensing into wireless EV charging infrastructure for reliable foreign object detection.

## 1. Introduction

Wireless power transfer technology is increasingly being adopted for electric vehicle charging systems due to its ability to provide convenient, automated, and cable-free charging. Compared with conventional plug-in charging methods, wireless charging improves user experience and enables advanced capabilities such as autonomous charging and bidirectional power transfer [1]. Apart from these advantages, ensuring operational safety remains a critical challenge in WPT systems [2].

One of the most important safety concerns in wireless EV charging is the presence of foreign conductive objects within the magnetic field between the transmitting and receiving coils. Such objects can experience significant heating due to induced eddy currents, potentially leading to safety hazards or system damage [3]. Conventional foreign object detection methods often rely on impedance monitoring, visual or infrared cameras, thermal sensing, or feedback from the receiver coil [4]. However, these approaches introduce high mechanical complexity and have limitations, particularly when the secondary coil is not present or when environmental conditions affect sensor measurements.

Industry standards such as SAE International J2954 require that safety verification must be performed both before the initiation of power transfer and continuously during power transfer. Therefore, reliable and robust detection mechanisms are essential for the practical deployment of wireless charging systems [5].

Magnetic Induction Tomography (MIT) has recently emerged as a promising technique for non-contact electromagnetic sensing. MIT systems utilise arrays of inductive coils to measure perturbations in electromagnetic fields caused by conductive materials [6,7]. A planar MIT sensing array PCB consisting of 16 air-core cylindrical coils arranged in a 4 × 4 configuration was used in [6]. The coils were mounted on a non-conductive PCB substrate, with dimensions of 18 × 18 cm and thickness t = 0.3 cm, with a spacing of 0.3 cm between adjacent coils. This MIT system had 120 unique coil pairs, giving a total of 120 independent measurements per frame. This configuration enabled spatial electromagnetic sensing of the charging region [6]. The sensing array PCB used in this study was based on the MIT measurement coil arrangement presented in [6], although the dimensions used in the present study differ from those reported in the reference. The sensing system used in this work was based on a Magnetic Induction Tomography (MIT) architecture developed for electromagnetic field imaging and foreign object detection, for which patent protection has been applied [8].

It should be noted that alternative system configurations may employ different sensing geometries depending on the WPT coil topology. For example, higher-power systems or topologically expanded WPT coils, such as dual-pole or tri-pole transmitter structures, may incorporate sensing arrays with different coil counts or spatial layouts. Although the hardware configuration may vary, the underlying sensing principle and data processing methodology remain functionally equivalent.

However, there has thus far been limited investigation of real-time foreign object detection using MIT-based machine learning systems under practical EV charging conditions [9]. MIT-based sensing generates high-dimensional electromagnetic measurements that are difficult to analyse using conventional signal processing methods. In addition, sensor drift, environmental variations, and electromagnetic interference can significantly affect detection performance. Recent advances in machine learning provide powerful tools for analysing complex sensor signals and identifying patterns associated with foreign objects [10].

In this paper, a machine learning-based foreign object detection system using MIT sensor data is proposed. The approach incorporates feature engineering techniques that extract statistical, temporal, and frequency-domain characteristics from electromagnetic sensor measurements to improve robustness against sensor drift and environmental variability.

The proposed system enables:

- Foreign object detection in wireless EV charging systems under no power transfer conditions.
- Robust operation under sensor drift and environmental variations.
- Real-time detection using MIT sensor arrays.
- Preliminary validation of the proposed foreign object detection system under active wireless power transfer conditions.

The main contributions of this work are summarised as follows:

Development of a large experimental dataset of 17,800 labelled frames for MIT-based foreign object detection, collected across a wide range of object types, positions, orientations, and multi-object configurations in accordance with SAE J2954.

Design of a feature-engineering pipeline that extracts 13 statistical, temporal, and frequency-domain features from MIT measurement frames, improving real-time detection robustness against inter-session sensor drift and environmental variation compared to raw sensor values.

Comprehensive evaluation of four machine learning classifiers—Random Forest, XGBoost, SVM, and MLP—under both offline validation and real-time cross-session deployment.

Preliminary validation of the proposed system under active wireless power transfer conditions, with further under-power testing and dataset expansion identified as ongoing work.

The remainder of this paper is organised as follows: Section 2 reviews the related work; Section 3 describes the hardware setup of the MIT sensing system; Section 4 details the proposed methodology and feature engineering approach; Section 5 explains the data acquisition process; Section 6 presents the data preprocessing techniques used to improve signal quality; Section 7 describes the overall system architecture; Section 8 presents and discusses the experimental results, including offline validation, real-time deployment, and preliminary under-power testing; Section 9 concludes this paper; and, finally, Section 10 outlines directions for future work.

## 2. Related Work

### 2.1. Wireless Power Transfer for Electric Vehicle Charging

Wireless power transfer technology has seen rapid development and adoption for electric vehicle charging applications over recent years, driven by demand for convenient, cable-free, and autonomous charging solutions [1]. Magnetically coupled resonant WPT systems have demonstrated efficient energy transfer over practical air gaps, enabling deployment in both static and dynamic EV charging infrastructure. Notwithstanding these advances, ensuring operational safety remains a central challenge, as the strong alternating electromagnetic field generated between the transmitter and receiver coils poses a significant risk when conductive foreign objects are present [4]. Industry standards, including SAE J2954, mandate that FOD must be performed both before the initiation of power transfer and continuously during operation, establishing a clear safety requirement that drives the research presented in this work [5].

### 2.2. Foreign Object Detection in Wireless Power Transfer Systems

Foreign object detection has been investigated extensively, given its critical safety functionality in WPT systems. Coil-based detection techniques monitoring variations in mutual inductance and magnetic field distribution have been proposed to identify specifically metallic objects in charging environments [11,12]. Electromagnetic model-based methods estimate deviations from expected coupling parameters to detect hazardous conditions introduced by foreign objects during operation [13]. Detection coil optimisation and frequency-based sensing approaches have been explored to improve sensitivity and reliability, analysing changes in resonant frequency and self-inductance caused by conductive materials near the charging pad [14,15]. The influence of metallic foreign objects on WPT system parameters, including coupling coefficient and power transfer efficiency, has been characterised in [16], providing physical motivation for the sensing approach adopted in this work. Impedance and load monitoring for foreign object detection has also been investigated in [4]. Despite these advances, conventional coil-based methods typically rely on single-point or limited spatial measurements and struggle under coil misalignment and environmental variability and in scenarios where the secondary coil is absent.

Alternative FOD approaches, such as camera-based and thermal imaging systems, have been explored for wireless EV charging applications. While these methods can achieve useful detection performance, they introduce significant hardware complexity and cost, require unobstructed line-of-sight to the charging pad, and are sensitive to ambient lighting conditions, outdoor temperature variation, and vehicle undercarriage occlusion. Furthermore, such systems are inherently single-function and require separate hardware subsystems for coil alignment and living object detection, further increasing overall system complexity and cost.

The planar MIT architecture employed in this work addresses these limitations by consolidating foreign object detection, living object detection, and coil alignment into a single compact PCB-mounted inductive array using low-cost air-core coils driven by a microcontroller. Unlike optical and thermal approaches, the MIT system operates passively before and during power transfer without requiring line-of-sight access and is unaffected by ambient lighting or surface contamination. This makes the proposed system a substantially simpler and more cost-effective solution for combined FOD, LOD, and alignment compared to existing imaging-based architectures.

### 2.3. Magnetic Induction Tomography for Electromagnetic Sensing

Magnetic Induction Tomography provides spatially distributed non-contact electromagnetic sensing by measuring field perturbations induced by conductive materials in a multi-coil array. The foundational planar MIT architecture used in this work was developed in [6], demonstrating 3D near-subsurface electromagnetic imaging using an air-core coil array on a planar PCB substrate. Reviews of MIT sensing methods and applications are provided in [8]. Three-dimensional MIT implementations for practical depth imaging in low-conductivity bodies have been reported in [17], and multi-frequency MIT systems for metallic object imaging have been demonstrated in [18]. The specific MIT architecture employed in this work is protected under the patent described in [9], which was developed for FOD, live object detection, and alignment applications in WPT systems. Recent advances in MIT hardware design and signal processing have further expanded the applicability of MIT sensing to practical embedded systems.

### 2.4. Machine Learning Approaches for Foreign Object Detection

Machine learning has recently emerged as a powerful approach for improving FOD reliability beyond the capability of threshold and model-based methods. A cost-effective sensor-free ML-based method for metallic object detection in WPT systems using decision tree models was demonstrated in [19], showing that data-driven classifiers can achieve reliable detection without additional hardware. A dual-modality approach combining machine vision with auxiliary detection coils to reduce false positives was proposed in [20]. Random Forest classifiers [19] have demonstrated strong generalisation on structured sensor data with limited training samples and provide built-in feature importance measures relevant to this work. XGBoost [20], with its built-in regularisation, has achieved strong performance on structured tabular classification tasks and is well-suited to deployment under distributional shift conditions.

### 2.5. Research Gap and Motivation

Even considering recent progress, several critical limitations remain unaddressed in the literature. Most existing ML-based FOD approaches operate on raw sensor measurements sensitive to drift and environmental variation, and few evaluate performance under realistic cross-session deployment conditions that expose this degradation. No existing study combines a spatially distributed planar MIT array with a feature engineering pipeline specifically designed to provide inter-session drift robustness in a WPT FOD context. The majority of published systems report only same-session offline accuracy, which significantly overstates real-world performance. This work directly addresses these gaps by proposing and validating a drift-robust feature engineering methodology for MIT-based FOD, evaluated under both offline and real-time cross-session deployment conditions, with preliminary validation under active power transfer also presented.

## 3. Hardware Setup

**Figure 1:**
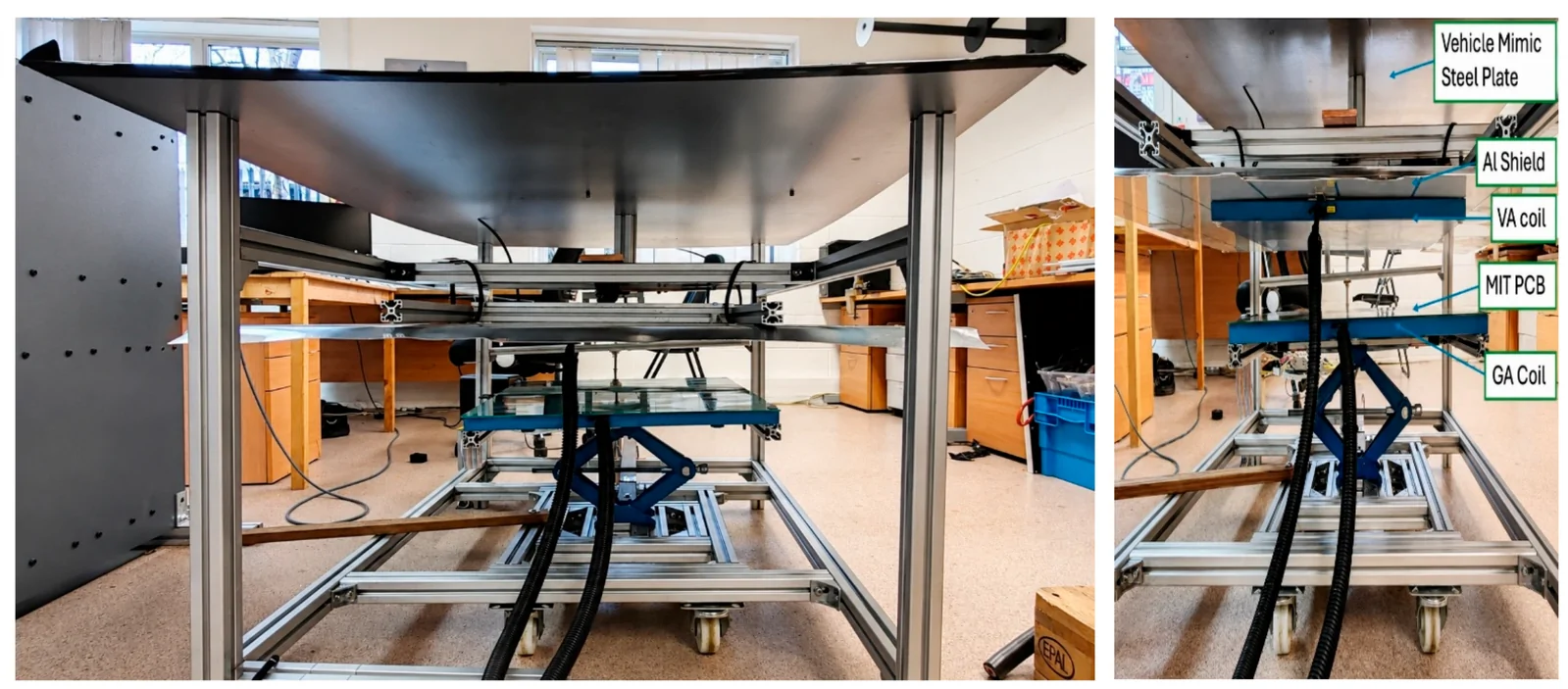
WPT test bench experimental setup, showing the primary coil assembly, secondary coil, and 6-axis positioning rig constructed in accordance with SAE J2954 Section 15.1.1.

The primary and secondary coils used for power transfer were mounted on a custom-built test rig that allowed six-axis positional adjustment between the transmitting and receiving coils. The experimental setup is shown in Figure 1. An aluminium (Al) shield was positioned above the Vehicle Assembly (VA) coil in accordance with the test standard, and a steel vehicle mimic plate was used to represent the smallest electric vehicle specified in the standard. This adjustability enabled systematic evaluation across a range of coil alignments and air gaps representative of real-world EV charging scenarios. The test rig was constructed in accordance with Section 15.1.1 of the SAE J2954 standard to ensure that the data collected under this configuration closely matched actual deployed operating conditions.

**Figure 2:**
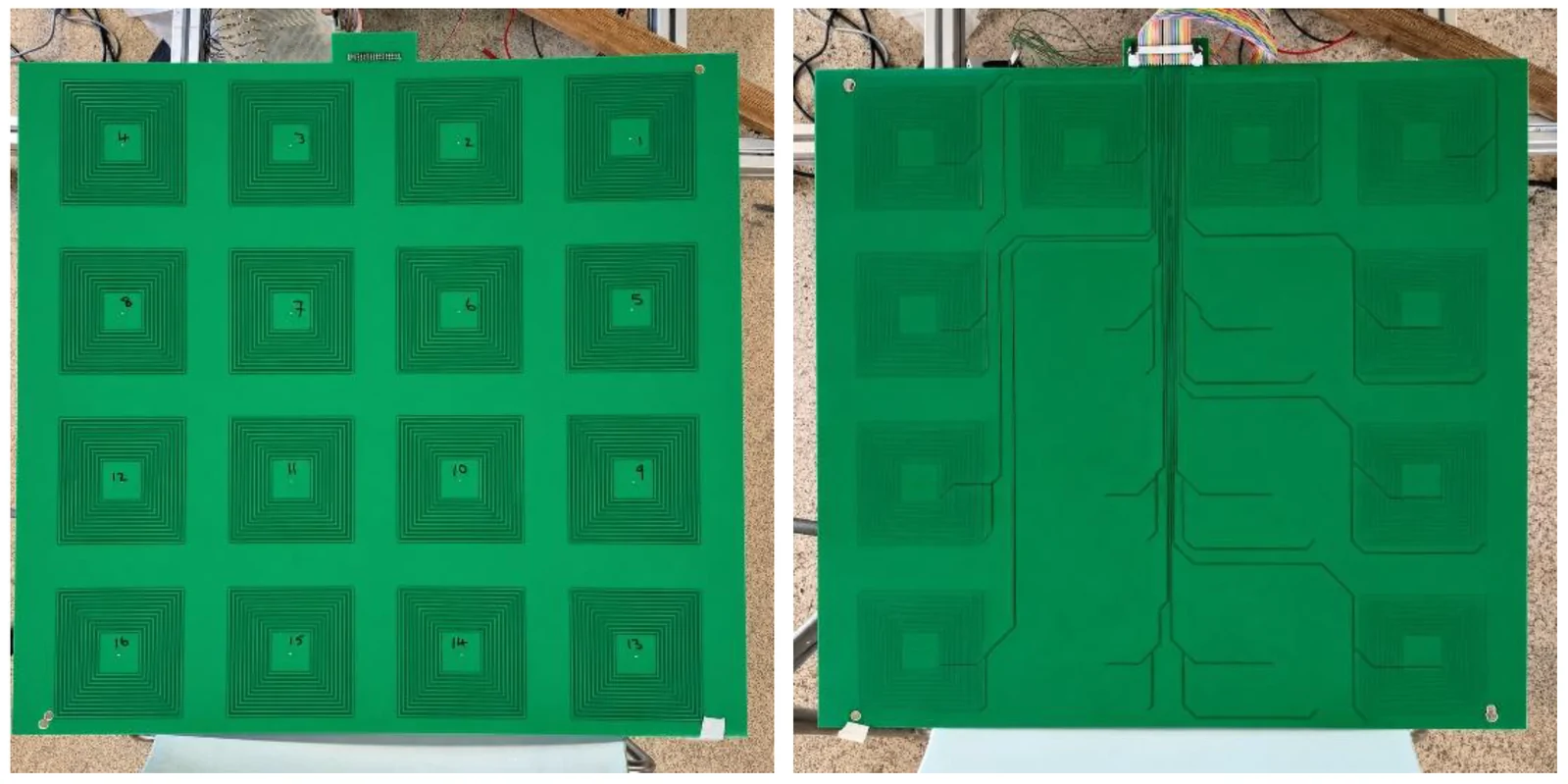
Planar MIT sensing PCB (front and rear), showing the 4 × 4 array of 16 air-core square coils arranged on the non-conductive PCB substrate.

The MIT sensing array was a two-layer PCB manufactured on a 1.6 mm substrate. The front layer carried the sensing coil array, while the rear layer carried the inter-coil signal routing traces and via interconnects. The front and rear images of the MIT sensing PCB can be seen in Figure 2.

**Figure 3:**
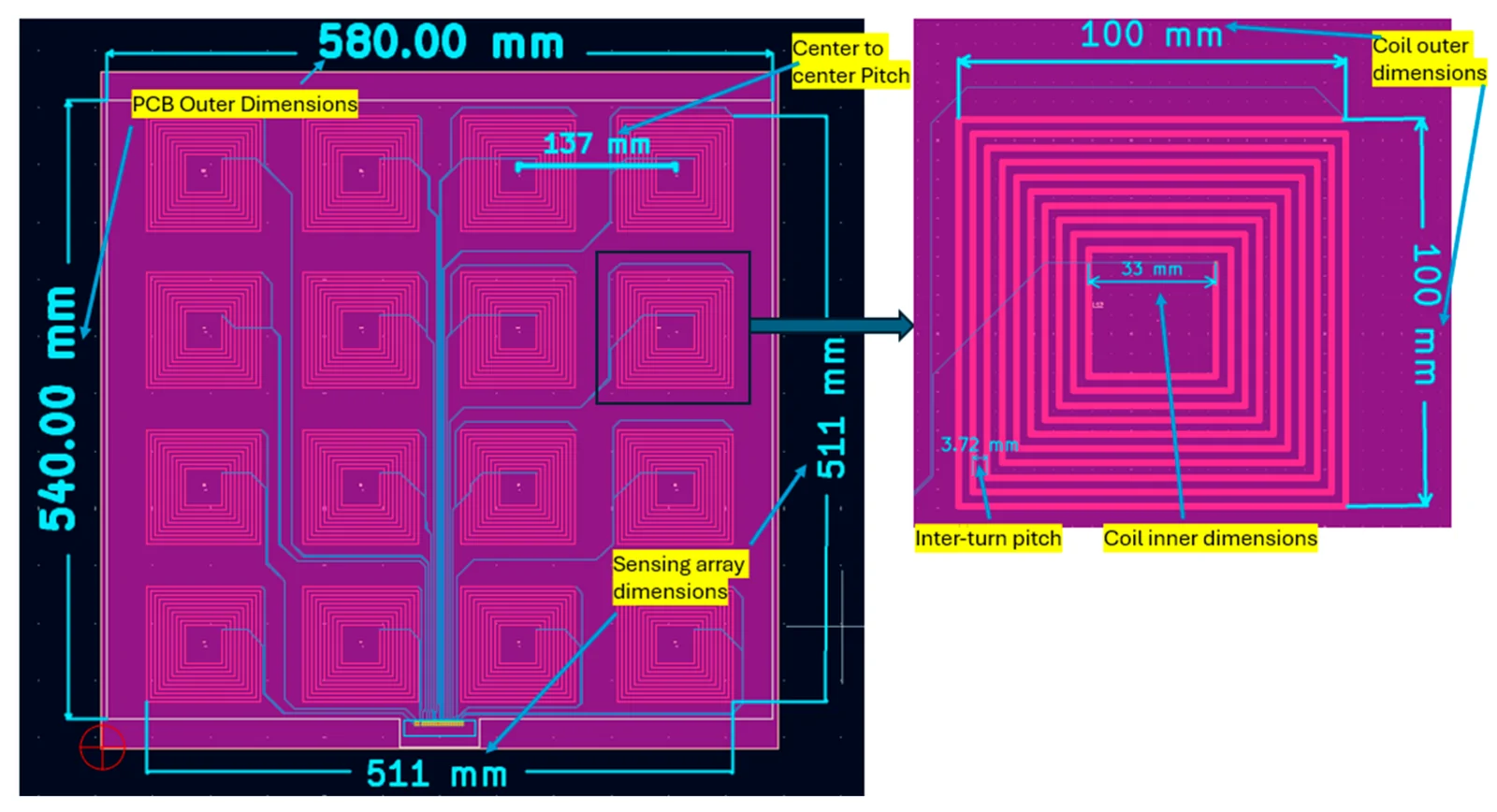
CAD model of the MIT measuring coil, with key dimensions.

The sensing array consisted of 16 air-core square coils arranged in a 4 × 4 grid, with a centre-to-centre pitch of 137 mm in both axes. Each coil was a 10-turn square spiral with outer dimensions of 100 mm × 100 mm and inner dimensions of 33 mm × 33 mm. The coil traces were 1.6 mm wide with an inter-turn pitch of 3.72 mm. The gap between the outer edges of adjacent coils was 37 mm. The full PCB measured 580 mm × 540 mm, with the active sensing array occupying a footprint of 511 mm × 511 mm. The PCB was secured on top of the primary coil via non-conductive nylon bolts to avoid any metallic fastener interference with the electromagnetic measurements. The dimensions and structure of the MIT sensing PCB are shown in Figure 3.

**Figure 4:**
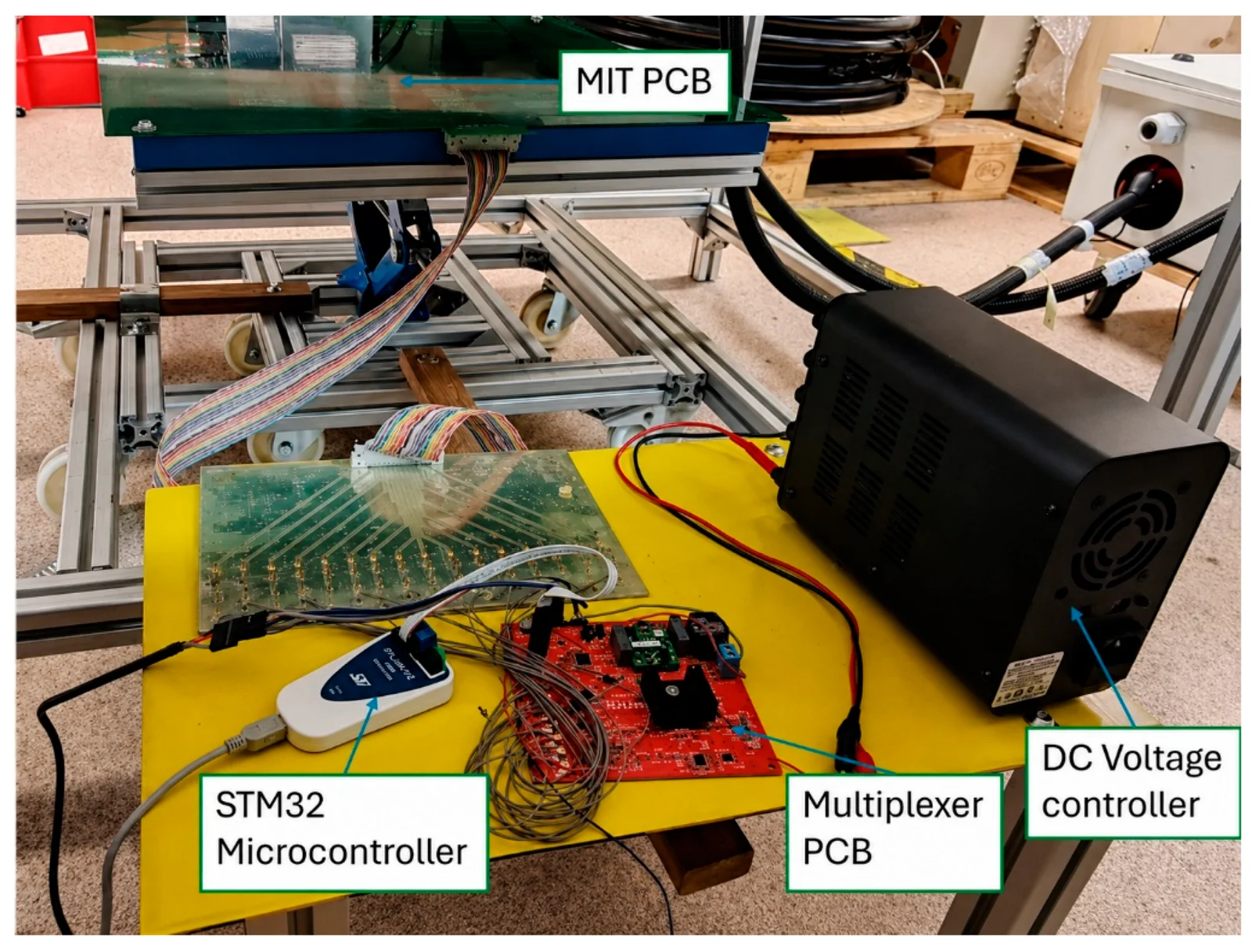
Data collection setup showing the MIT sensing array PCB mounted on the primary coil and the STM32 microcontroller interface unit.

The MIT array PCB interfaced with an STM32G431K8T6 microcontroller system (STMicroelectronics, Geneva, Switzerland), responsible for sequentially exciting coil pairs, sampling the resulting electromagnetic responses, and transmitting the measurement frames to a host computer via a serial connection. Firmware programming and debugging were performed using an ST-LINK/V2-ISOL programmer (STMicroelectronics, Geneva, Switzerland). The data collection setup is shown in Figure 4.

## 4. Methodology

The proposed system performed foreign object detection (FOD) in wireless power transfer environments using Magnetic Induction Tomography (MIT) sensor data and machine learning models. The methodology consisted of four main stages: sensor data acquisition, signal preprocessing, feature engineering, and machine learning-based classification.

The overall objective was to learn a mapping from electromagnetic sensor measurements to the presence of any conductive object:

(1) f:x→y

where x ∈ R120 represents the vector of sensor readings from the MIT array and *y* ∈ {0, 1} denotes the class label (non-conductor or conductor).

### 4.1. Sensor Data Acquisition

The MIT sensing PCB consisted of multiple inductive coils arranged in a grid. During each measurement cycle, the system recorded electromagnetic responses from the sensing region. Let

(2) $$x=\left[x_{1},x_{2},x_{3},\ldots ,x_{120}\right]$$

denote a single measurement frame, where *xᵢ* represents the electromagnetic response captured from the *i*-th sensing channel. Each frame corresponds to the spatial electromagnetic state of the charging region at a given time.

### 4.2. Signal Preprocessing

Sensor readings may contain noise and outliers caused by environmental disturbances such as electromagnetic interference, temperature variation, or measurement instability. To mitigate these effects, a median-based outlier correction method was applied.

First, the median of the measurement frame was computed:

(3) $$m=\mathrm{median}\left(x_{1},x_{2},\ldots ,x_{120}\right)$$

A value was considered an outlier if

(4) $$\left|x_{i}-m\right|>\tau m$$

where τ is a deviation threshold (10% in this study). Outlier values were replaced using

(5) $$x_{i}^{*}=\left\{\begin{array}{cc} x_{i}, & if\;\left|x_{i}-m\right|\leq \;\tau m\\ m, & otherwise\; \end{array}\right.$$

To further smooth the signal, a Savitzky–Golay filter was applied:

(6) $$\hat{x_{i}}=\sum_{k=-K}^{K}c_{k}x_{i+k}$$

where *cₖ* are polynomial smoothing coefficients. This process reduced high-frequency noise while preserving signal characteristics relevant for detection.

### 4.3. Feature Engineering

Directly using raw sensor signals can lead to poor generalisation due to sensor drift and environmental changes. Therefore, the proposed system extracted statistical, temporal, and frequency-domain features from each measurement frame. Let the processed signal be

(7) $$\hat{x}=\left[\hat{x_{1}},\hat{x_{2}},\ldots ,\hat{x_{120}}\right]$$

The engineered feature vector was defined as

(8) $$z=\varphi \left(\hat{x}\right)$$

where φ(·) denotes the feature extraction function.

#### 4.3.1. Statistical Features

Features such as mean, standard deviation, median, skewness, and kurtosis summarise the overall distribution of sensor readings. These descriptors capture structural patterns in a signal rather than relying on individual measurements, making them less sensitive to noise and drift. The statistical properties of the signal were computed as follows.

The mean was

(9) $$\mu =\frac{1}{N}\sum_{i=1}^{N}\hat{x_{i}}$$

The standard deviation was

(10) $$\sigma =\sqrt{\frac{1}{N}\sum_{i=1}^{N}{\left(\hat{x_{i}}-\mu \right)}^{2}}$$

The range was

(11) $$R=\max\left(\hat{x_{i}}\right)-\min\left(\hat{x_{i}}\right)$$

The root mean square was

(12) $$\mathrm{RMS}=\sqrt{\frac{1}{N}\sum_{i=1}^{N}\hat{x_{i}^{2}}}$$

Mean and RMS reflect overall signal energy, while standard deviation captures variability. Skewness indicates asymmetry in a signal distribution caused by conductive disturbances and kurtosis identifies sharp signal peaks associated with object presence. Higher-order statistics such as skewness and kurtosis are also calculated to characterise the distribution of a signal.

#### 4.3.2. Temporal Features

The argmax and argmin indices provide temporal information about when a signal reaches extreme values. This is particularly useful in MIT systems where conductive materials alter the timing of the electromagnetic response. The mean absolute derivative measures how rapidly a signal changes, reflecting how quickly a system reacts to the presence of conductive objects. Temporal dynamics of the signal were captured using derivative-based features.

The mean absolute derivative was

(13) $$D=\frac{1}{N-1}\sum_{i=1}^{N-1}\left|\hat{x_{i+1}}-\hat{x_{i}}\right|$$

and the indices of extreme responses were

(14) $$i_{max}=argmax_{i}\left(\hat{x_{i}}\right),\;\;\;\;i_{min}=argmin_{i}\left(\hat{x_{i}}\right)$$

These features provided information about how the electromagnetic field changed across the sensing array.

#### 4.3.3. Frequency-Domain Features

The fast Fourier transform (FFT) features played a crucial role in differentiating between conductor and non-conductor conditions.

Frequency characteristics were extracted using Fast Fourier transform (FFT):

(15) $$X\left(k\right)=\sum_{n=0}^{N-1}\hat{x_{n}}\;e^{-\frac{j2\pi kn}{N}}$$

The magnitudes of the dominant spectral components were used as features:

(16) $$F_{k}=\left|X\left(k\right)\right|$$

The magnitudes of the first three non-zero FFT components represented dominant frequency characteristics of the signal. These frequency signatures were influenced by:

- Material conductivity;
- Eddy current distribution;
- Energy absorption characteristics.

In this work, the first three non-zero frequency components were selected: F_1_, F_2_, F_3_.

These features captured variations in electromagnetic energy distribution caused by conductive objects.

The final feature vector was, therefore,

(17) $$z=\left[\mu ,\sigma ,\mathrm{median},R,\mathrm{skewness},\mathrm{kurtosis},\mathrm{RMS},i_{max},i_{min},D,F_{1},F_{2},F_{3}\right]$$

Experimental observations indicated that these FFT-based features provided consistent separability between classes, even under varying environmental conditions and sensor drift.

**Figure 5:**
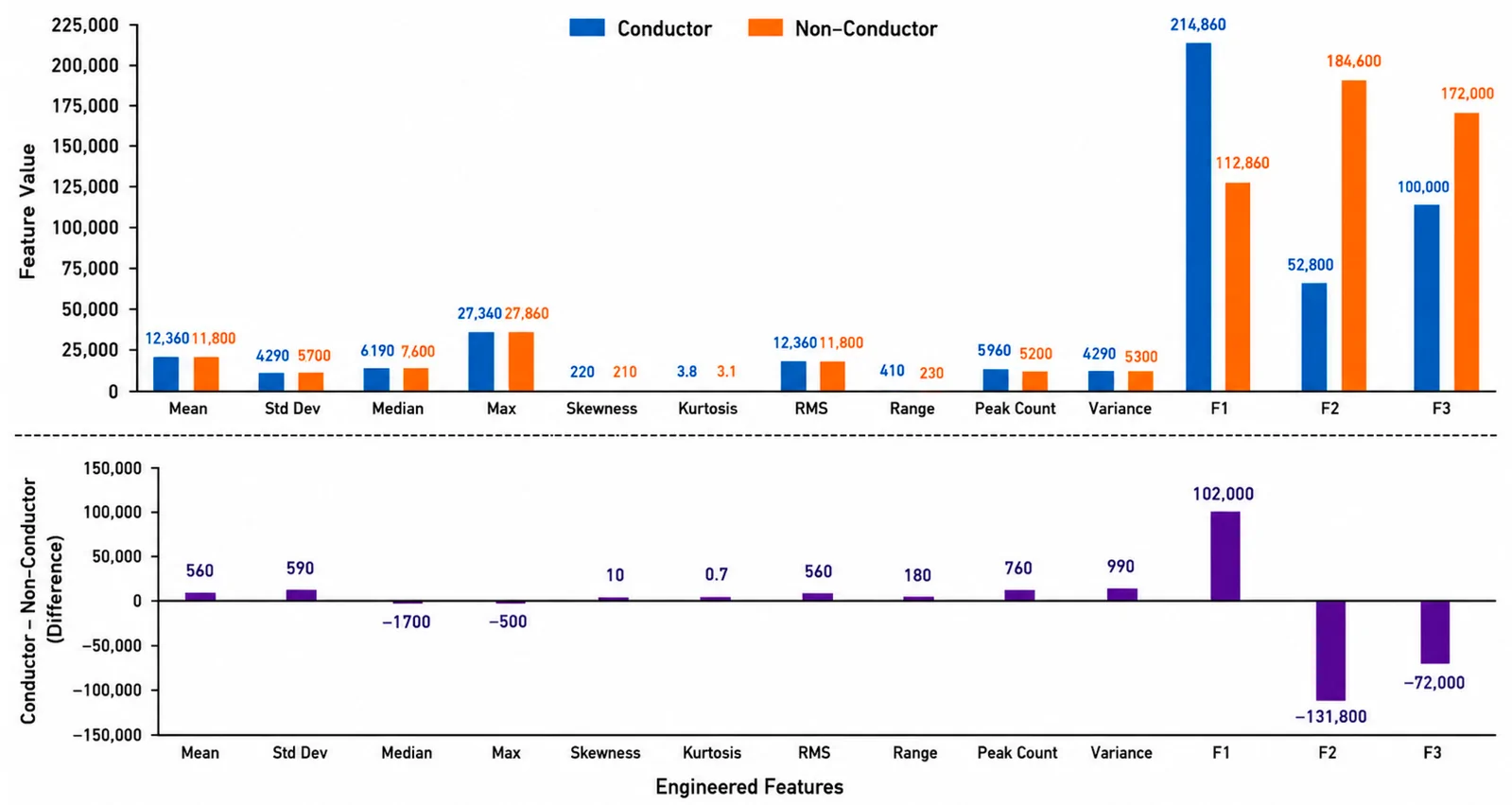
Comparison of engineered features extracted from sensor readings under conductor and non-conductor conditions. The upper panel shows the feature values for each condition, while the lower panel illustrates the difference (conductor—non-conductor) for each feature. Positive values indicate higher values for conductors, whereas negative values indicate higher values for non-conductors.

Among all engineered features, the frequency-domain features (11)–(13) demonstrated the highest discriminative power during experimentation. This can be seen in Figure 5.

### 4.4. Feature Selection Rationale

The 13 features in the final vector, z = [μ, σ, median, R, skewness, kurtosis, RMS, i_max_, i_min_, D, F1, F2, F3], were selected based on three criteria: physical interpretability, discriminability between conductor and non-conductor classes, and robustness to environmental conditions.

Statistical features (μ, σ, median, R, skewness, kurtosis, RMS) described the spatial distribution of responses across the 120-channel frame. When a conductor was present, eddy currents created a non-uniform energy distribution across the array, increasing spread, asymmetry, and peak sharpness relative to the no-object condition. Because these features described within-frame relative patterns rather than absolute signal levels, they were less sensitive to the uniform baseline shifts caused by inter-session drift.

Temporal features (i_max_, i_min_, D) identified which channels responded most strongly and how sharply the response changed across adjacent channels, providing spatial and geometric information about the object’s position and the extent of it within the sensing region.

Frequency-domain features (F1, F2, F3) were the magnitudes of the first three non-zero FFT components of the spatial measurement vector and consistently demonstrated the highest discriminative power in the experiments.

The dominance of Fourier transform components as the most discriminative features in this system has a direct physical explanation rooted in the spatial structure of the MIT measurement frame.

Each measurement frame was a 120-element vector x = [x_1_, x_2_, …, x_120_], where each element represented the electromagnetic mutual inductance response from one of the 120 unique coil pairs in the 4 × 4 MIT array. This vector was not a time series; it was a spatial map of the electromagnetic coupling state across the sensing region at a single instant. Applying the FFT to this spatial vector decomposed the energy distribution across the array into spatial frequency components, analogous to how a 2D FFT decomposes an image into spatial frequency content.

When no object was present, the electromagnetic response was relatively uniform across the sensing array, producing a flat spatial distribution dominated by a single low-spatial-frequency component with low energy at higher spatial frequencies. When a conductive object was introduced, eddy currents were induced in the object, and these distorted the electromagnetic field in a spatially structured, object-dependent way. This distortion created a localised perturbation in the spatial measurement vector, a region of elevated or suppressed coupling responses concentrated around the coil paired nearest to the object. This localised spatial perturbation had a characteristic frequency signature in the FFT domain: the first non-zero spectral component F1 reflected the dominant low-spatial-frequency energy associated with the bulk presence of a conductor across the array, while F2 and F3 captured the finer spatial structure related to object size, geometry, and position within the sensing region.

### 4.5. Machine Learning-Based Classification

The foreign object detection task was formulated as a supervised classification problem. Given a dataset,

(18) $$D=\{\left(z_{i},y_{i}\right){\}}_{i=1}^{M}$$

where $z_{i}\in R^{13}$ is the engineered feature vector and $y_{i}\in \{0,\;1\}$ denotes the class label (non-conductor or conductor), the goal was to learn a classifier $h\left(z\right)$ that mapped features to predicted labels $\hat{y}$ :

(19) $$\hat{y}=h\left(z\right)$$

Four machine learning models were evaluated: Random Forest, Support Vector Machine, eXtreme Gradient Boosting (XGBoost), and Multi-Layer Perceptron.

#### 4.5.1. Random Forest (RF)

Random Forest is an ensemble of T decision trees $\left\{h_{t}{\}}_{t=1}^{T}\right.$ . Each tree predicts a label for an input feature vector, z , and the final prediction is obtained via majority voting:

(20) $$\hat{y}=\mathrm{mode}\{h_{t}\left(z\right){\}}_{t=1}^{T}$$

where $h_{t}(z)$ is the prediction from the t -tree.

#### 4.5.2. Support Vector Machine (SVM)

SVM constructs a hyperplane that maximises the margin between classes using a kernel function, $K(z_{i},z_{j})$ . The decision function is

(21) $$\hat{y}=\mathrm{sign}\left(\sum_{i=1}^{N}\alpha_{i}y_{i}K\left(z_{i},z\right)+b\right)$$

where $\alpha_{i}$ are Lagrange multipliers, b is the bias term, and K(·) is typically a linear or RBF kernel.

#### 4.5.3. eXtreme Gradient Boosting (XGBoost)

XGBoost is an ensemble of K regression trees $\{f_{k}{\}}_{k=1}^{K}$ . The objective function combines a loss term and a regularisation term:

(22) $$L=\sum_{i=1}^{N}l\left(y_{i},\hat{y_{i}}\right)+\sum_{k=1}^{K}\Omega \left(f_{k}\right)$$

where $l(y_{i},{\hat{y}}_{i})$ is the classification loss (e.g., log-loss) and $\Omega (f_{k})$ penalises the complexity of the tree $f_{k}$ to avoid overfitting. The model prediction is

(23) $$\hat{y_{i}}=\sum_{k=1}^{K}f_{k}\left(z_{i}\right)$$

#### 4.5.4. Multi-Layer Perceptron (MLP)

MLP is a feedforward neural network. For an input z , the activation of the layer l is

(24) $$a^{\left(l\right)}=\sigma \left(W^{\left(l\right)}a^{\left(l-1\right)}+b^{\left(l\right)}\right)$$

where $W^{\left(l\right)}$ and $b^{\left(l\right)}$ are the weights and biases of the layer l , $a^{\left(0\right)}=z$ , and σ is a nonlinear activation function (ReLU). The output layer uses a SoftMax function to predict class probabilities:

(25) $$\hat{y}=\mathrm{softmax}\left(a^{\left(L\right)}\right)$$

### 4.6. Algorithm Selection Rationale

Four supervised classification algorithms were selected for evaluation: Random Forest (RF), Support Vector Machine (SVM), eXtreme Gradient Boosting (XGBoost), and Multi-Layer Perceptron (MLP). Each model was evaluated under two conditions—raw sensor values (120 MIT measurements per frame, no feature-engineering) and feature-engineered values (13 statistical, temporal, and frequency-domain features)—to quantify the direct impact of the proposed feature engineering approach on classification performance.

The four algorithms were chosen because they collectively represent the major families of supervised learning: bagging ensembles (RF), margin-based classifiers (SVM), boosting ensembles (XGBoost), and neural networks (MLP). This diversity ensured that any observed performance trends—particularly the improvement from raw to feature-engineered inputs—reflected a general property of the feature representation rather than the behaviour of a single model class.

Random Forest was selected for its robustness to correlated and noisy inputs and its ability to generalise from limited training data. SVM with an RBF kernel provides a well-established non-linear baseline that is effective with both high-dimensional raw inputs (120 features) and reduced engineered feature spaces (13 features). XGBoost was included due to its strong empirical performance on structured tabular data and its regularisation terms, which help prevent overfitting under the sensor drift conditions observed in real-time deployment [17]. MLP served as a neural network baseline to determine whether deep representation learning added value over the hand-crafted features at the scale of this dataset.

Evaluating all four models, on both raw and feature-engineered inputs, allowed for a direct, controlled comparison that isolated the contribution of feature engineering independently of model choice. If the improvement held consistently across all four model families, this provided strong evidence that the feature engineering methodology—rather than any particular classifier—was responsible for the observed gains in robustness and real-time accuracy.

Deep learning approaches such as CNNs and LSTMs were not included as the primary input was a compact scalar feature vector rather than raw time series or image data; this will be explored in future work with larger datasets collected under active power transfer conditions.

## 5. Data Acquisition

Data were collected under four conditions: conductive materials, multiple conductive materials, non-conductive materials, and no-object scenarios. Objects were placed at various positions on top of the MIT sensing PCB to capture spatial variability in sensor responses.

The selection of test objects followed the standard SAE J2954, which defined a representative set of foreign objects for evaluating minimum FOD capability. As stated in the standard, although the range of possible foreign objects is effectively unlimited, a predefined set of test objects ensures repeatability, safety relevance, and consistency across testing environments. The materials used in this study were selected in accordance with Section 16.2.2 of the standard [5]. Table 1 shows the recommended objects and their alignment as per the SAEJ2954 standards. In this study, the recommended objects as well as other objects are used.

In addition to the recommended objects, further conductive materials were included to enhance dataset diversity. Objects included aluminium bars, bronze bars, steel bolts, coins, copper bars, threaded steel rods, metal cans (with and without liquid), and batteries. Scenarios involving multiple conductive objects and conductive materials placed over non-conductive materials were also considered.

For conductive materials, 13,900 sets of sensor readings were collected. For non-conductive conditions (including no-object cases), approximately 3900 sets were recorded using materials such as cardboard, plastic, cloth, and mixed non-conductive objects. Each data sample consisted of 120 numerical sensor readings, corresponding to a full measurement frame from the MIT system. Multiple samples were collected under identical conditions to reduce random measurement variability and improve dataset reliability.

Beyond object type, several additional parameters were systematically varied during data collection to ensure that the dataset captured the full range of conditions encountered in a real wireless EV charging environment. Each conductive and non-conductive object was placed at multiple spatial positions across the MIT sensing PCB, including at the centre, edges, and corners, to account for the positional dependence of the electromagnetic response. Objects were also tested in different orientations, such as flat, tilted, and upright, given that the angle and surface area of a conductive object relative to the coil plane directly influenced the magnitude and spatial distribution of the induced eddy currents captured by the sensor array. In scenarios involving multiple simultaneous objects, their relative spacing and arrangement were also varied. For each unique combination of object, position, and orientation, 25 consecutive sets of sensor readings were collected. This repetition served two purposes: first, it provided sufficient samples for the median filtration step described in Section 5, which required a minimum set size to reliably identify and replace outlier readings; second, it captured short-term temporal variability in the sensor output under static conditions, improving the statistical representativeness of each recorded condition. The total dataset of 17,800 frames, therefore, reflected variation across object type, material conductivity, spatial position, orientation, multi-object configuration, and acquisition session, rather than object identity alone.

The data acquisition process was implemented using a Python-based interface communicating with an STM32 microcontroller via a serial connection operating at 115,200 baud. For each measurement cycle, the Python script sent a command to trigger data transmission from the embedded system.

Each measurement frame consisted of 240 bytes, representing 120 sensor values. The system included a validation mechanism whereby incomplete data frames were discarded and automatically reacquired to ensure data integrity. The validated data were stored in a structured format, where each column represented a single acquisition instance and each row corresponded to an individual sensor channel.

The collected dataset was subsequently exported for further signal processing and machine learning analysis. While the current implementation relied on a Python-based interface, future system development will integrate this functionality into a fully embedded real-time processing framework.

Data were also collected during active wireless power transfer to evaluate system behaviour under electromagnetic interference. These measurements were acquired during a 4 kW power transfer scenario, following the same data acquisition procedure described above. A total of 300 samples were collected for conductive conditions and 300 samples for non-conductive conditions.

It should be noted that the primary focus of this study was the development of a reliable foreign object detection system before the initiation of power transfer. Consequently, the dataset collected under active power transfer conditions was comparatively smaller. Ongoing work aims to expand this dataset and develop a robust detection framework specifically for operation during power transfer.

## 6. Data Preprocessing

The collected data showed occasional fluctuations. These outliers were replaced with the median value to reduce noise. Certain readings displayed pronounced spikes, which could be effectively mitigated using median filtering. For each condition, 25 sets of numerical data were collected, and median filtration was applied to remove these anomalies.

**Figure 6:**
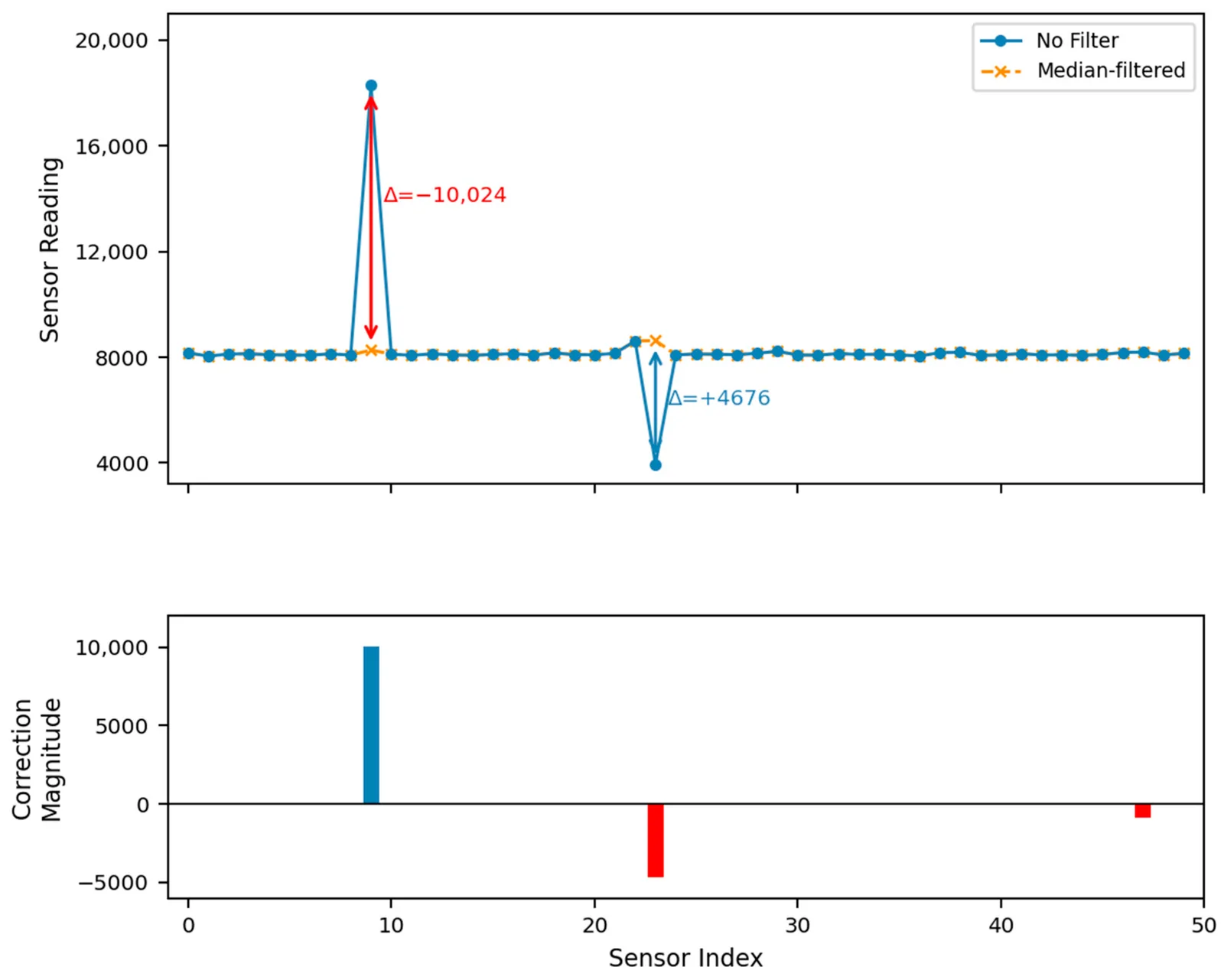
Effect of median-based outlier correction on sensor readings. The upper plot compares unfiltered and median-filtered readings across a 50-sample measurement frame, where red arrows indicate correction events, with the corresponding magnitude (Δ) annotated at each site. The lower plot shows the correction magnitude at each sensor index, confirming that the filter selectively corrected only samples exceeding the deviation threshold τ = 10%, while leaving all remaining readings unchanged.

Figure 6 illustrates the comparison between sensor readings with and without median filtering.

Spikes in sensor readings, possibly caused by external factors, resulted in abnormally high or low values. Median filtration was implemented via a Python script that identified values deviating more than 10% from the median value and replaced them with the overall median of the dataset. The same approach was applied to live sensor data used for prediction, where isolated abnormal readings were replaced by the median.

Since the data consisted of complete numerical frames, there were no missing values, and abnormal extremes were effectively addressed. Additionally, RandomOverSampler was used to balance the classes, ensuring that the model learned equally from conductor and non-conductor cases. Finally, Savitzky–Golay filtering was applied to smooth the data and assess its impact on prediction accuracy.

A key challenge in real-world deployment is the gradual shift in baseline MIT readings between sessions caused by temperature changes, humidity, and low-frequency electromagnetic interference. This causes models that achieve near-perfect offline accuracy on stored data to perform poorly when deployed across different sessions without recalibration.

The system addressed drift at two levels. At the signal level, median filtering removed impulsive spikes and Savitzky–Golay filtering suppressed high-frequency noise. At the feature level, the engineered features, particularly statistical descriptors such as standard deviation, skewness, and kurtosis, characterised the relative spatial distribution of readings within each frame rather than their absolute values. Since drift manifested as a near-uniform shift across all 120 channels, the within-frame spatial distribution remained largely stable, making these features inherently more drift-robust than raw sensor values. The evaluation of system performance under controlled coloured noise and active power transfer EMI conditions is identified as a priority for future work.

## 7. System Architecture

**Figure 7:**
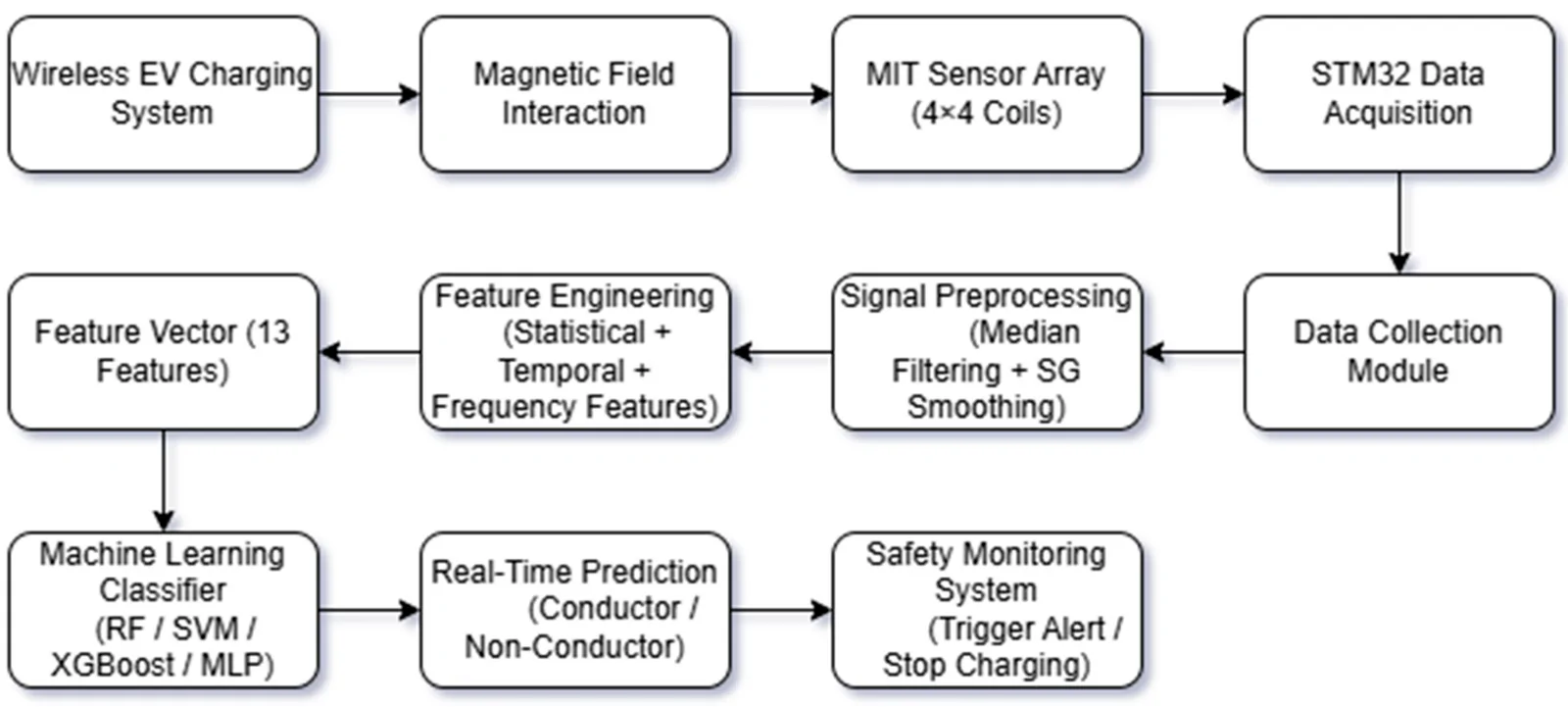
System architecture of the foreign object detection system.

The overall architecture of the proposed foreign object detection system is illustrated in Figure 7. The system integrates a magnetic sensing framework with machine learning-based classification to detect conductive foreign objects in a wireless electric vehicle (EV) charging environment.

When objects were present within the charging region, they interacted with the electromagnetic field. Conductive objects in particular induced eddy currents that distorted the magnetic field distribution.

These disturbances were captured by a planar Magnetic Induction Tomography (MIT) sensor array. The sensor array measured variations in the magnetic field caused by the presence of nearby materials. Each coil pair generated electromagnetic measurements representing the coupling characteristics between transmitting and receiving coils.

The acquired analogue signals were then digitised using an STM32-based data acquisition module, which recorded the electromagnetic measurements in real time. The collected data were stored by the data collection module, forming a dataset of measurement frames corresponding to different object conditions.

Before analysis, the raw signals underwent signal preprocessing to improve data quality and reduce measurement noise. In this work, a combination of median filtering and Savitzky–Golay (SG) smoothing was applied to remove impulsive noise while preserving important signal characteristics.

Following preprocessing, feature engineering was performed to transform the processed signals into a compact and informative representation. Statistical, temporal, and frequency-domain features were extracted from the signals, producing a feature vector consisting of 13 features for each measurement frame. These features captured key characteristics of the electromagnetic response associated with conductive and non-conductive objects.

The resulting feature vectors were then provided as inputs to several machine learning classifiers, including Random Forest (RF), Support Vector Machine (SVM), XGBoost, and Multi-Layer Perceptron (MLP). These models learned patterns in the electromagnetic measurements and classified each sample as either a conductor or a non-conductor.

The trained models were subsequently used for real-time prediction, enabling rapid identification of foreign conductive objects within the charging region. When a conductive object was detected, the system activated the safety monitoring module, which could trigger alerts or automatically stop the charging process to prevent overheating or potential hazards.

**Figure 8:**
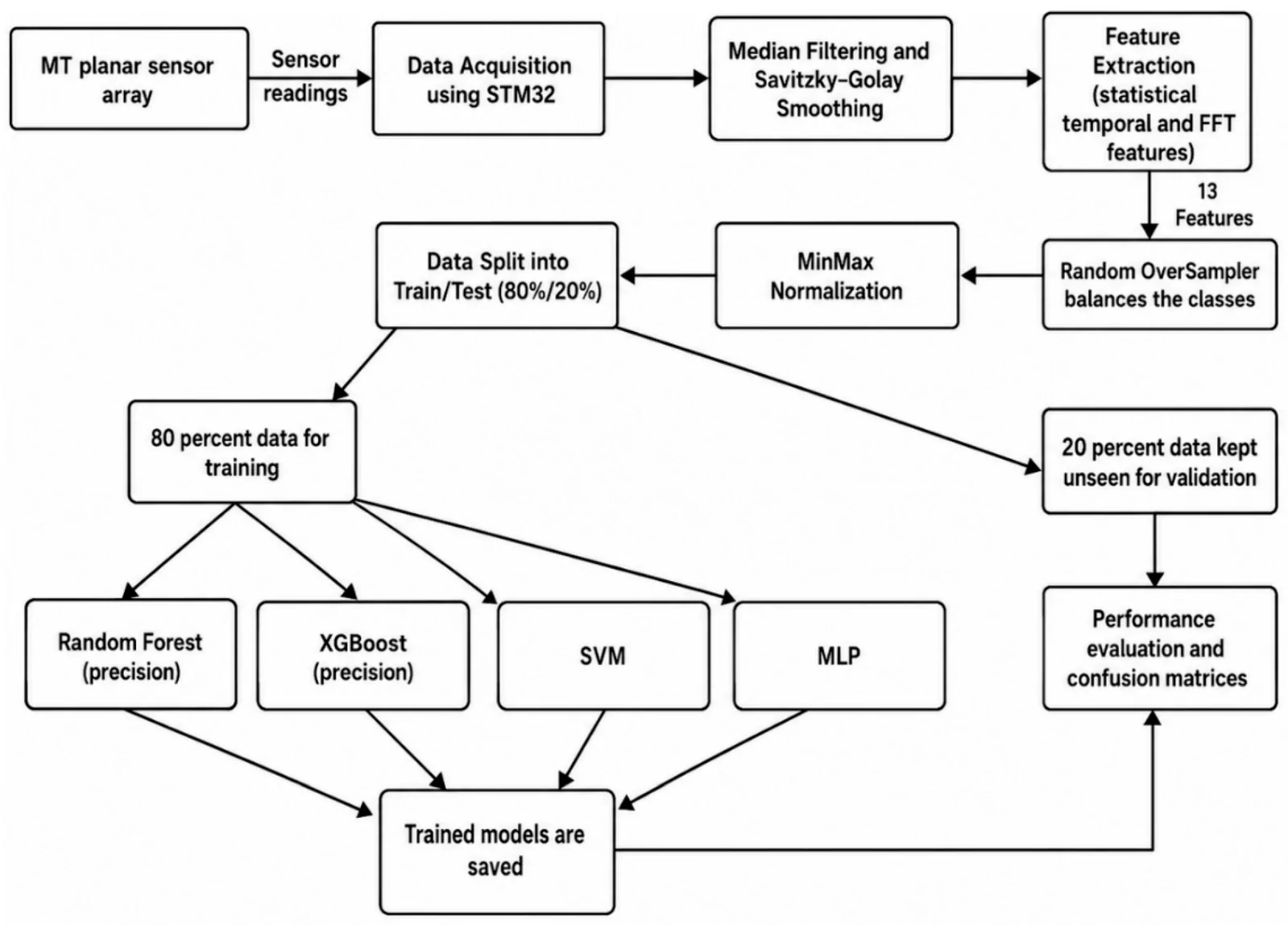
Computational procedure of the system and ML pipeline.

This architecture enables a reliable and automated framework for foreign object detection in wireless EV charging systems, combining electromagnetic sensing with machine learning-based decision making. The computational procedure is shown in Figure 8.

## 8. Results

**Figure 9:**
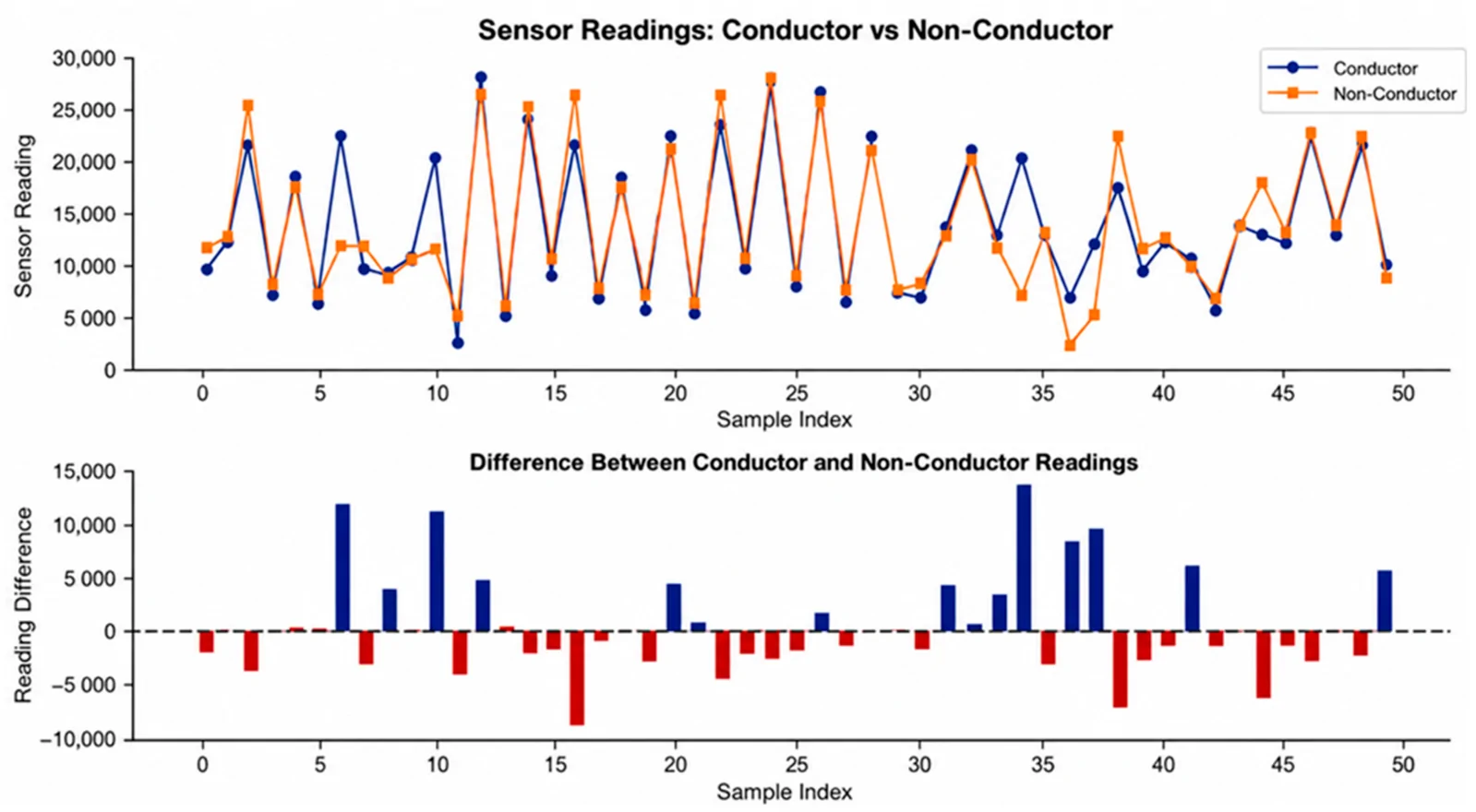
This line plot illustrates the variation in sensor readings obtained when a conductor and a non-conductor were introduced near the sensing coil. Each point represents a measurement captured during sequential sampling, highlighting the difference in response between the two material conditions. The accompanying difference plot shows the point-wise difference between the conductor and non-conductor readings, emphasising the magnitude and direction of deviation across the measurement set.

A noticeable difference was observed in the sensor response when a conductive object was present within the electromagnetic field compared to when the sensing region was empty (see Figure 9). The graph shows the variation in the sensor readings when a small conductive object was introduced into the field. This distinction allowed multiple machine learning models to achieve high performance during offline evaluation. When trained and validated using the predefined train–test split and during cross-validation of the collected dataset, most models consistently achieved accuracies exceeding 95%.

In the above figure, the difference in sensor readings while introducing a small conductive object (50p GBP coin) is shown. This forms the reason for the very high accuracy during validation on the collected set of data.

However, a challenge emerged when deploying the models in real-time conditions. The sensor readings were affected by sensor drift, environmental variations (temperature, humidity, electromagnetic interference), and temporal changes in system characteristics. As a result, the prediction accuracy in real-time scenarios dropped compared to the offline evaluation results. To mitigate this, an experimental manual calibration procedure was introduced, which demonstrated the capacity to recover detection accuracy; however, it required frequent manual intervention, making it impractical for deployment. This limitation was subsequently addressed through the introduction of a feature engineering pipeline, which is discussed in detail in the following section.

Table 2 presents the validation results for all four models evaluated on raw, non-feature-engineered sensor values (120 MIT sensor readings per frame) under four filtering conditions: no filter, Savitzky–Golay filtering (SGF), median filtering, and combined median + SGF. The table reports five performance metrics for each configuration. Accuracy, precision, recall, and F1 score were computed on the held-out 20% offline test partition using stratified sampling from the collected dataset. The real-time accuracy column reports the number of correct predictions, out of 50 attempts recorded, during live deployment sessions conducted on separate days from the training session, where the model received new sensor frames directly from the physical MIT hardware without any recalibration. This metric was stricter than offline accuracy because the deployment frames were subject to inter-session sensor drift, ambient temperature variation, and electromagnetic interference not present during data collection. The confusion matrix columns—true positive (TP), true negative (TN), false positive (FP), and false negative (FN)—are reported for the offline test set and provide a detailed breakdown of classification errors per configuration.

As shown in Table 2, all four models achieved very high offline accuracy on raw sensor values, with Random Forest, MLP, and XGBoost reaching 99–100% accuracy and F1 scores of 0.99–1.00 under median + SGF filtering and SVM reaching 98% under the same condition. The near-zero false positive and false negative counts under median filtering confirm that the raw sensor data contained a strong class-separable signal when evaluated on the same-session stored dataset. However, the real-time accuracy figures tell a substantially different story: without feature engineering, correct predictions in live deployment ranged from 21 to 31 out of 50 real-time prediction attempts across all model and filter configurations, corresponding to 42% to 62% real-time accuracy. This large gap between offline and real-time performance, up to 38 percentage points for some configurations, directly demonstrates the impact of inter-session sensor drift on raw sensor values and motivated the feature engineering approach evaluated in Table 3. Notably, even the best-performing non-FE configuration (Random Forest, median + SGF, 62% real-time accuracy) fell well below the minimum reliability threshold expected for a safety-critical FOD system, confirming that signal filtering alone is insufficient to address drift without feature-level robustness.

In addition, to address this issue, an experimental calibration mechanism was investigated. In this approach, a baseline dataset representing the no-object condition was first established. During real-time deployment, the system initiated a calibration phase in which the user verified that no object was present in the sensing region. New sensor readings were then collected and compared with the baseline measurements. If significant deviations were detected, the model was retrained or adjusted before performing predictions. This calibration-based approach improved real-time prediction performance, achieving a manually measured accuracy of approximately 90%. However, the need for frequent recalibration made the method impractical for continuous real-world deployment.

The real-time accuracy was computed as

(26) $$\frac{Correct\;Predictions}{Total\;number\;of\;predictions}\times 100$$

Table 3 presents the validation results for the same four models evaluated under the same four filtering conditions, but now using the proposed feature-engineered (FE) input representation. The 13-element feature vector comprising statistical, temporal, and frequency-domain components was extracted from each raw MIT measurement frame, as described in Section 6. All other experimental conditions, including the train/test split, cross-validation procedure, and real-time deployment protocol, were identical to those used to generate Table 2, enabling a direct, controlled comparison between the two input representations.

The offline validation results in Table 3 remained consistently high across all model and filter configurations, with Random Forest and XGBoost achieving 99–100% accuracy and F1 scores of 0.98–1.00, MLP reaching 99% under median-based filtering, and SVM achieving 97% under the same conditions. The confusion matrix entries confirm near-zero false positive and false negative counts for the best configurations, with Random Forest and XGBoost (Median + SGF) recording 0 FP and 0 FN on the offline test set, identical to the non-FE results in Table 2. This indicates that offline separability between conductor and non-conductor classes was strong under both input representations when evaluated on the same-session data.

The critical distinction between Table 2 and Table 3 lies in the real-time accuracy. With feature-engineering applied, real-time correct predictions increased substantially across every model and filter configuration. Random Forest and XGBoost, with combined median + SGF filtering, each achieved 49/50 correct predictions (98% real-time accuracy), compared with 62% and 60%, respectively, for the same models without feature engineering under the same filtering conditions, a gain of 36–38 percentage points. MLP improved from 58% to 86% under the same filtering conditions and SVM improved from 56% to 88%, representing gains of 28 and 32 percentage points, respectively. These consistent cross-model gains confirm that the improvement is attributable to the feature engineering methodology rather than to any specific classifier.

Even without any filtering, the feature-engineered models substantially outperformed their non-FE counterparts in real-time conditions: Random Forest achieved 86% (43/50) versus 48% (24/50) and XGBoost achieved 88% (44/50) versus 48% (24/50). This demonstrates that feature engineering alone—independent of the filtering pipeline—provided a significant baseline improvement in real-time robustness by extracting drift-invariant representations from the raw sensor data. The further gains observed when median filtering and SGF were added confirm that the two mechanisms—feature-level drift invariance and signal-level noise suppression—were complementary rather than redundant.

Among all evaluated configurations, Random Forest and XGBoost with median + SGF filtering and feature engineering achieved the highest real-time accuracy of 98% and are identified as the recommended deployment configuration for the proposed FOD system. SVM showed a notable performance gap relative to the tree ensemble methods (88% vs. 98% real-time accuracy) despite comparable offline accuracy, consistent with its known sensitivity to absolute feature scaling under distributional shift. MLP, while showing improvement over its non-FE baseline, reached a maximum real-time accuracy of 86%, suggesting that at the scale of this dataset, the neural network did not offer additional generalisation benefits over the ensemble methods. These findings collectively validate the central hypothesis of this work: that a feature engineering approach combining statistical, temporal, and frequency-domain representations of MIT sensor data can substantially mitigate the effects of inter-session sensor drift and environmental variation, enabling reliable, real-time foreign object detection without requiring manual recalibration between deployment sessions.

### 8.1. Real-Time Performance Evaluation

To evaluate the real-time performance of the proposed system, a controlled experimental validation procedure was conducted. The validation involved 10 conductive objects and 10 non-conductive objects. Among these, five objects from each category were used during training, while the remaining five from each category were previously unseen by the model, ensuring that the evaluation directly tested the generalisation capability of the trained classifiers under realistic deployment conditions.

The end-to-end prediction pipeline operated as follows. The STM32 microcontroller acquired a complete 240-byte measurement frame (120 sensor values) via serial communication at 115,200 baud. Upon receipt, the Python-based inference script applied the preprocessing step—median-based outlier replacement and, where applicable, Savitzky–Golay filtering—in under 5 ms. Feature extraction from the processed frame, where applicable, added a further 3–5 ms. The trained classifier then performed inference in under 10 ms on the host platform, yielding a total end-to-end latency of approximately 75–80 ms per prediction cycle. This corresponded to a sustained prediction rate of approximately 5 predictions per second during real-time operation.

During testing, each object was placed at different spatial locations within the sensing region. After each test, the object was removed, and a no-object condition was verified and recorded before the next trial. A total of 50 experimental conditions were tested for each model and filter configuration, with accuracy computed as the number of correct predictions out of 50 attempts.

The results are summarised in Table 4. Across all models and filter configurations, the feature-engineered (FE) pipeline substantially outperformed the non-feature-engineered (non-FE) baseline, confirming that the proposed feature engineering approach was the primary driver of real-time robustness rather than the choice of classifier alone.

Without feature-engineering, correct predictions ranged from 21 to 31 out of 50 (42–62%), reflecting the sensitivity of raw sensor values to drift and environmental variation between the training and deployment sessions. With feature engineering applied, correct predictions increased to 39–49 out of 50 (78–98%) across all models.

Among the evaluated configurations, Random Forest and XGBoost with combined median + Savitzky–Golay filtering achieved the highest real-time accuracy, with each recording 49 correct predictions out of 50 (98%). These two models are therefore recommended as the deployment configuration for the proposed system. The MLP achieved 43/50 (86%) under the same filtering conditions, while SVM reached 44/50 (88%), both representing acceptable performance but falling below the tree-ensemble methods. Notably, the addition of median filtering alone consistently produced a larger accuracy gain than SGF alone across all models, suggesting that impulsive outlier removal is the more critical preprocessing step for real-time robustness, with SGF providing a secondary smoothing benefit when combined with median filtering.

### 8.2. Validation Under Active Power Transfer Conditions

To address the critical operational requirement that an FOD system must function reliably during active wireless power transfer, a supplementary validation experiment was conducted with the WPT system operating under power. During active power transfer, the primary coil generated a strong alternating electromagnetic field at the operating frequency, introducing significant EMI into the MIT sensing array that was absent under the no-power-transfer condition. This represented a more challenging operational condition relevant to scenarios considered in SAE J2954, although the present evaluation was limited to a controlled experimental setup.

For this validation, 300 measurement frames were collected for each class—conductive and non-conductive—with the WPT system actively transferring power. Data collection, preprocessing, and inference followed the same pipeline described in Section 4, Section 5, and Section 6. Only the two best-performing model configurations identified in Section 8.1 and Section 8.2—Random Forest and XGBoost—were evaluated, under both no-filter and combined median + SGF filtering conditions. The results for non-feature-engineered and feature-engineered inputs are presented in Table 5 and Table 6, respectively.

As shown in Table 5, both Random Forest and XGBoost achieved high accuracy on raw non-feature-engineered values under active power transfer, reaching 99% accuracy and zero false positives and false negatives under combined median + SGF filtering. This suggests that the preprocessing pipeline helps reduce the impact of periodic EMI introduced by the active WPT field at the signal level.

Table 6 shows that the feature-engineered pipeline achieved comparable performance, with both models reaching 98–99% accuracy under median + SGF filtering, with very low misclassification. The marginal reduction in accuracy for the no-filter feature-engineered condition (96–97%) relative to the no-filter non-feature-engineered condition (97–98%) is consistent with the observation that the FFT-based features partially captured WPT carrier frequency components in the absence of filtering, which were suppressed once median and SGF filtering were applied. Across all evaluated configurations, the system maintained above 96% accuracy under active power transfer, indicating that the proposed MIT-based FOD approach showed promising robustness to electromagnetic interference generated during active wireless power transfer under the tested conditions.

It should be noted that the results during active power transfer are based on a limited set of controlled laboratory conditions and a finite dataset size; further validation across broader operating environments, hardware variations, and extended testing durations will be performed in future to fully establish generalisability. Future work will focus on developing the object detection system during power transfer.

## 9. Conclusions

This study proposed and evaluated a machine learning-based foreign object detection system for wireless EV charging using a planar Magnetic Induction Tomography sensor array. The system combines a custom STM32-based data acquisition module with a feature-engineering pipeline that extracts 13 statistical, temporal, and frequency-domain features from 120-channel MIT measurement frames, enabling robust classification of conductive and non-conductive objects under realistic operating conditions.

A dataset of 17,800 labelled measurement frames was collected across a wide range of object types, positions, orientations, and multi-object configurations in accordance with SAE J2954. Four classification models (Random Forest, XGBoost, SVM, and MLP) were evaluated under both raw and feature-engineered input representations across four filtering conditions. Offline validation results consistently exceeded 97% accuracy across all models and filter configurations for both input types. A key finding of this work is the behaviour under real-time cross-session deployment: without feature-engineering, real-time accuracy fell to 42–62% across all models due to inter-session sensor drift and environmental variation. With the proposed feature engineering applied, real-time accuracy increased to 78–98%, with Random Forest and XGBoost under combined median + SGF filtering each achieving 98% accuracy, a gain of 36–38 percentage points over the non-feature-engineered baseline.

To address the operational requirement of FOD during active wireless power transfer, a supplementary validation experiment was conducted with the WPT system actively transferring power. Both Random Forest and XGBoost maintained above 96% accuracy across all filter configurations under active power transfer conditions, reaching 98–99% efficiency under combined median + SGF filtering with near-zero misclassification. These results confirm that the proposed system is robust to the strong electromagnetic interference generated during active charging.

The proposed approach eliminates the need for frequent manual recalibration, is implementable on low-cost embedded hardware, and satisfies the spatial sensing requirements that single-coil impedance methods cannot meet.

## 10. Future Work

Several directions are identified to further develop the proposed system. Firstly, the under-power validation presented in Section 8.1 used 300 samples per class, collected at a single power level. Future work will expand this to multiple power levels and operating frequencies consistent with the full SAE J2954 power class range and will include a systematic characterisation of system performance as a function of WPT field intensity to establish operating boundaries.

Secondly, the current evaluation addresses binary conductor/non-conductor classification. Future work will extend this to multi-class object categorisation, distinguishing object material, size, and type, which would provide richer information for safety management decisions beyond simple detection.

Thirdly, the robustness of the system under controlled coloured noise conditions, including 1/f pink noise and band-limited interference at the WPT switching frequency, will be evaluated to quantify the noise margins of the feature engineering pipeline and inform the design of adaptive filtering strategies.

Fourth, adaptive learning techniques will be investigated to enable continuous model updating as sensor characteristics evolve over the system lifetime, further reducing sensitivity to long-term drift without manual intervention.

Fifth, the dataset will be expanded to include a wider variety of object geometries, composite materials, and environmental conditions, including wet surfaces and temperature extremes, to further assess generalisation to the full range of objects encountered in deployed EV charging infrastructure.

Finally, deep learning approaches, including convolutional and recurrent neural networks, will be evaluated on larger datasets collected under active power transfer conditions, to determine whether automatic feature learning from raw or minimally processed MIT frames can match or exceed the performance of the feature engineering pipeline proposed in this work.

## Figures and Tables

**Table 1:** Table of recommended test objects as per SAEJ2954 [5].

Sample Objects	Alignment of the Test Object	Notes
Paper stack with paper clip	Largest parallel field	Five sheets of paper, at least 2 inches square, attached to a steel wire paper clip approximately 1.25 inches long. Location and orientation should refer to the paper clip. Stack assumed to be lying flat on the surface.
Foil with paper backing	Largest perpendicular field	2 × 4 inches, similar to a chocolate bar wrapper or cigarette foil material. Lying flat on the surface.
Coins	Largest perpendicular field	U.S. 5¢ coin.
Nail	Largest parallel field	10d common steel nail, coated.
Aluminum foil	Largest perpendicular field	2–3 inch square or circular piece, 0.002–0.010 inch thick.
Steel bar	Largest perpendicular field and largest parallel field	4 × 2.75 × 0.4 inches lying flat on the surface.

**Table 2:** Comparison of machine learning algorithms with different signal filtering strategies: validation metrics and real-time classification performance. All while using non-feature-engineered values.

ML Model	Filter Used	Validation on Already Collected Data				Real Time Accuracy	TP	TN	FP	FN
		Accuracy	Precision	Recall	F1 Score					
RF	No Filter	99	99	98	99	48	2780	808	12	0
	SGF	99	99	99	99	54	2780	814	6	0
	Median	99	100	99	99	54	2780	820	0	0
	SGF + Median	100	100	100	100	62	2780	820	0	0
MLP	No Filter	98	99	94	96	42	2778	778	42	2
	SGF	98	98	95	97	50	2780	783	37	0
	Median	99	99	99	99	52	2780	820	0	0
	SGF + Median	100	100	100	100	58	2780	820	0	0
SVM	No Filter	97	94	92	93	46	2780	756	64	0
	SGF	98	100	92	96	46	2780	762	58	0
	Median	98	100	92	96	50	2780	762	58	0
	SGF + Median	98	100	95	96	56	2780	762	58	0
XGBOOST	No Filter	99	99	99	99	48	2778	812	8	2
	SGF	99	99	99	99	56	2780	819	1	0
	Median	99	100	99	99	54	2780	820	0	0
	SGF + Median	99	100	99	99	60	2780	820	0	0

**Table 3:** Comparison of machine learning algorithms with different signal filtering strategies: validation metrics and real-time classification performance while using feature-engineered values.

ML Model	Filter Used	Validation on Already Collected Data				Real Time Accuracy	TP	TN	FP	FN
		Accuracy	Precision	Recall	F1 Score					
RF	No Filter	99	99	96	98	86	2776	789	31	4
	SGF	99	99	97	98	90	2780	795	25	0
	Median	99	100	99	99	92	2780	819	1	0
	SGF + Median	100	100	100	100	98	2780	820	0	0
MLP	No Filter	98	98	93	95	78	2774	759	61	6
	SGF	98	98	93	95	80	2769	778	42	11
	Median	99	99	99	99	80	2780	818	2	0
	SGF + Median	99	99	99	99	86	2780	820	0	0
SVM	No Filter	95	89	89	89	80	2697	738	82	93
	SGF	95	92	87	89	82	2720	727	93	60
	Median	97	96	92	94	86	2767	778	42	13
	SGF + Median	97	96	92	94	88	2775	783	37	5
XGBOOST	No Filter	99	99	96	97	88	2774	795	25	6
	SGF	99	99	97	98	92	2778	800	20	2
	Median	99	100	99	99	92	2780	817	3	0
	SGF + Median	99	99	99	99	98	2780	820	0	0

**Table 4:** Real-time prediction validation across models and preprocessing configurations.

Model Used	Type of Filter	Correct Predictions/50 Attempts	
		Non-FE Values	FE Values
Random Forest	No Filter	24	43
	SGF	27	45
	Median	27	46
	SGF + Median	31	49
Multi-Layer Perceptron	No Filter	21	39
	SGF	25	40
	Median	26	40
	SGF + Median	29	43
Support Vector Machine	No Filter	23	40
	SGF	23	41
	Median	25	43
	SGF + Median	28	44
XGBoost	No Filter	24	44
	SGF	28	46
	Median	27	46
	SGF + Median	30	49

**Table 5:** Validation under active power transfer for non-feature-engineered values (300 samples per class).

ML Algorithm	Filter Used	Validation on Collected Unseen Data				TP	TN	FP	FN
		Accuracy	Precision	Recall	F1 Score				
Random Forest	No Filter	98	100	96	98	60	58	2	0
	SGF + Median	99	100	98	99	60	60	0	0
XGBoost	No Filter	97	98	96	97	59	58	2	1
	SGF + Median	98	99	99	99	60	60	0	0

**Table 6:** Validation under active power transfer for feature-engineered values (300 samples per class).

ML Algorithm	Filter Used	Validation on Collected Unseen Data				TP	TN	FP	FN
		Accuracy	Precision	Recall	F1 Score				
Random Forest	No Filter	96	98	95	96	59	57	3	1
	SGF + Median	99	98	97	99	60	60	0	0
XGBoost	No Filter	97	98	96	96	59	57	3	1
	SGF + Median	98	98	99	98	59	60	1	0

## Data Availability

Experimental data, as analysed and presented within this paper, is available on request. Further inquiries can be directed to the corresponding author.
